# Conceptual Models in Health Informatics Research: A Literature Review and Suggestions for Development

**DOI:** 10.2196/medinform.5021

**Published:** 2016-02-24

**Authors:** Kathleen Gray, Paulina Sockolow

**Affiliations:** ^1^Health and Biomedical Informatics CentreUniversity of MelbourneMelbourneAustralia; ^2^Health Systems & Sciences ResearchCollege of Nursing and Health ProfessionsDrexel UniversityPhiladelphia, PAUnited States

**Keywords:** medical informatics, theoretical models, conceptual framework, conceptual model, design-based research, implementation research, evaluation research, health informatics, research design, research training

## Abstract

**Background:**

Contributing to health informatics research means using conceptual models that are integrative and explain the research in terms of the two broad domains of health science and information science. However, it can be hard for novice health informatics researchers to find exemplars and guidelines in working with integrative conceptual models.

**Objectives:**

The aim of this paper is to support the use of integrative conceptual models in research on information and communication technologies in the health sector, and to encourage discussion of these conceptual models in scholarly forums.

**Methods:**

A two-part method was used to summarize and structure ideas about how to work effectively with conceptual models in health informatics research that included (1) a selective review and summary of the literature of conceptual models; and (2) the construction of a step-by-step approach to developing a conceptual model.

**Results:**

The seven-step methodology for developing conceptual models in health informatics research explained in this paper involves (1) acknowledging the limitations of health science and information science conceptual models; (2) giving a rationale for one’s choice of integrative conceptual model; (3) explicating a conceptual model verbally and graphically; (4) seeking feedback about the conceptual model from stakeholders in both the health science and information science domains; (5) aligning a conceptual model with an appropriate research plan; (6) adapting a conceptual model in response to new knowledge over time; and (7) disseminating conceptual models in scholarly and scientific forums.

**Conclusions:**

Making explicit the conceptual model that underpins a health informatics research project can contribute to increasing the number of well-formed and strongly grounded health informatics research projects. This explication has distinct benefits for researchers in training, research teams, and researchers and practitioners in information, health, and other disciplines.

## Introduction

### Conceptualizing Research in Health Informatics

There is consensus that the discipline of health informatics is characterized by the integration of elements from many other fields of knowledge. The components of health informatics, apart from the biomedical sciences, include computer science, information science, decision science, statistics, cognitive science, organizational theory, and others [1]. In essence, health informatics is “cross-training” between broadly defined information sciences and health sciences [2].

Ideally, research rests on “methodologies that capture the processes integral to applications, the users and the world in which the users function” [3]. However, a growing number of researchers who do not identify themselves as health informaticians are now doing research into the design, implementation, and evaluation of information and communication technologies in the health sector. This growth is fuelled by new technologies that reduce the health professionals’ barriers to application development, and also by the growing market for consumer technologies that are not subject to medical device regulations. For example, the current emergence of apps for mobile phones and the increased ease of programming these apps is said to "enable busy clinicians to develop simple mobile Web-based apps for academic, educational, and research purposes, without any prior knowledge of programming" [4]. The ensuing research appears in the journal and conference literature of a variety of fields including clinical specialties, health policy, information management, and software engineering, to name a few. This paper is aimed at researchers still in training, or practitioners new to the field who wish to align their work more strongly with the discipline of health informatics.

Taking a disciplined approach to health informatics research means operating across the component domains of expertise by using integrative conceptual models. A working definition of a conceptual model is that it is an explanation of the researcher’s thinking about the key constituents of the research problem, and why the whole problem is greater than the sum of its parts because of the way these interconnect and interact. In any field of knowledge, using a conceptual model to describe something about a subset or an aspect of the domain has value; that is, a conceptual model makes explicit the intended meaning of terms and concepts used and avoids ambiguity and misinterpretation. The terms conceptual framework and conceptual model are used interchangeably in the literature [5], and we use the latter throughout this paper. Health informatics conceptual models that connect the knowledge and thus explain the research in the language of two broad domains, health science and information science, can help to ensure that research is effective and has impact.

Too often, research on information and communication technologies in the health sector appears to miss either the health problem or the information technology problem. Some examples of missing the health problem are: a review of 55 heart failure risk computational models noted that few had been implemented in clinical practice [6]; a description of a technical solution to a perceived clinical problem omitted any mention of consultation with clinical experts [7]; and a description of a technical solution to a clinical issue did not fit the clinical workflow [8]. If the research does not capture the processes integral to both the world of health science and the world of information science, valuable efforts are expended developing applications that do not address the intended problem. Many innovations fail to achieve sustainability or other measures of success because “the current development of eHealth technology often disregards the interdependencies between technology, human characteristics, and the socioeconomic environment, resulting in technology that has a low impact in health care practices” [9]. Evaluation research too may fall short of offering key insights. For example, researchers who evaluated an electronic health record implementation using the Delone and McLean framework for evaluating generic information systems success recognized that they would have done better to develop a more health-specific approach to evaluation [10], and indeed other researchers had customized Delone and McLean for evaluating health-specific information systems [11].

### Recognizing Conceptual Models in Health Informatics Research

Researchers new to the field of health informatics need to learn how to work with its conceptual models. However, this may not be taught formally, and exemplars and guidance in the literature are sporadic and scattered. Possibly due to publication word limits, much of the published health informatics research conveys an absence of discussion, even a lack of awareness, about the importance of conceptual models. In addition, papers may mention a conceptual model approach without specifying how it came into existence.

Some descriptions of the development of conceptual models in specific health informatics research studies are available. Gordon et al described how models of clinical guideline knowledge had to be integrated with models of health care activities and processes in a conceptual model approach for automating distribution of clinical guidelines [12], and Ruland and Bakken enumerated the components of a conceptual model to support inclusion of patient preferences in clinical decision making and underscored the importance of incorporating knowledge from four domains [13]. In addition, Kaplan and Shaw compared a variety of ways that evaluation researchers have conceptualized the complex social and institutional dynamics of health information technology implementations [14], and Yusof et al [15] explained how theories from information science and evidence from health science informed the structure of a human-, organization-, and technology-fit evaluation framework for health information systems. There is no single right or wrong conceptual model that brings order to a set of ideas about a health informatics problem. The exercise of making the conceptual model explicit in a study, in words and/or figures, is critical to clarify what is known and to identify what is in question or not known, from the perspective of the researchers. Through the process of debating the merits of alternative conceptual models, their explanatory power, completeness, and other aspects about how well they represent the research objectives, new theories are formed.

This paper aims to promote more explicit use of health informatics conceptual models in research on information and communication technologies in the health sector, and encourage discussion of these conceptual models in scholarly forums. Learning how to develop and apply integrative conceptual models is an educational issue for researchers in training, and so too for those who train them and those who review their work. Using conceptual models has research significance for building the discipline of health informatics and benefits for many stakeholders in health informatics research.

## Methods

A two-part method was used to structure and illustrate ideas of how to work effectively with conceptual models in health informatics research. First, we conducted a search of the literature of conceptual models, and then we used a qualitative research process to formulate a step-by-step approach to developing a conceptual model.

We looked for papers published up to 2014 that described the development of conceptual models in health information science and technology research in PubMed, IEEE Xplore, ACM Digital Library, Scopus, and Web of Knowledge. First the Medical Subject Heading (MeSH) "models, theoretical" paired with "medical informatics" were used. Then search terms were widened to include "research design" (especially where there was discussion of why a design was chosen). An additional search looked for possible pairings of "design science" or "design-based research", "implementation", and "evaluation" with health information systems and technology and with electronic health (e-health). Selection of a cross-section of full papers that made substantial mention of conceptual models was based on reading abstracts and also on mining reference lists from selected papers for further examples.

Critical reflection was used to formulate a step-by-step approach to developing a conceptual model. We examined the assumptions embedded in our experiences, associated them with a range of different factors, re-evaluated them using external reference points, and re-worked our ideas and practices [16]. Specifically, we drew on our separate experiences working in multidisciplinary health information technology research teams internationally over five years. We reconsidered the bases of our expertise as researchers and also as reviewers, supervisors, advisors, and examiners of research in health informatics. We analyzed the literature we had retrieved, looking at ways authors named, explicated, and sequenced key components in the development of a conceptual model. We agreed on specific steps in development of a conceptual model and went back to the literature and to our experience repeatedly for examples.

Using this method, we produced a set of suggestions, which remains untested in terms of its technical validity and sufficiency. Nevertheless, after formulating these suggestions, we found that teaching novice researchers to use conceptual models in a step-wise manner is recognized as effective by academics in other fields [17]. We also found that a similar idea had appeared in the literature of a different field of health research, namely health program evaluation [18]. The existence of these peer-reviewed publications added external validity to our method.

## Results

The process of critical review and reflection led us to a consensus that supported a seven-step approach to developing a conceptual model for health informatics research. In this section we offer these steps and examples from the literature as a guide to the novice health informatics researcher. The methodology for working with conceptual models in health informatics involves (1) acknowledging the limitations of health science and information science conceptual models; (2) giving a rationale for one’s choice of an integrative conceptual model; (3) explicating a conceptual model verbally and graphically; (4) seeking feedback about a conceptual model from stakeholders in both the health science and information science domains; (5) aligning a conceptual model with an appropriate research plan; (6) adapting a conceptual model in response to new knowledge over time; and (7) disseminating conceptual models in scholarly and scientific forums.

### Acknowledge the Conceptual Models of Contributing Domains

It is important to acknowledge that both health science and information science use a variety of conceptual models to represent entities and relationships in their respective domains; however, these fields of knowledge are differentiated by their approaches to conceptualizing problems. A defining characteristic of any given health informatics research problem is how it responds to the challenge to acknowledge the applicability and also the inadequacy of conceptual models from both health science and information science. That is, you should be able to describe some parts of the research question using concepts that appropriately represent the problem to the separate audiences or stakeholders from each domain. You must also go beyond this, to frame the problem in a conceptual model that transcends and integrates these domains.

In the information sciences, a conceptual model may also be referred to as a domain model. Furthermore, conceptual modeling should not be confused with other modeling disciplines, such as data modeling, logic modeling, or physical modeling. Many different conceptual models may be used for demonstration, optimization, construction, simulation and other activities in the application domain [19]. In the field of information science, recent examples in the literature can be found that discuss specific conceptual models in detail [20-22]. The complexity of health is a major reason why health informatics is not just another application domain in information science [23]. In the health sciences, an introduction to a range of conceptual models for defining and conceptualizing health argues that simplistic definitions of health lead to equally simplistic measures of health, health outcomes, and quality of care [24]. In the field of health, recent examples which explore conceptual models can be found [25-28].

### Review Conceptual Models Already Used in Health Informatics

The next step is to review the health informatics literature for conceptual models that can be either applied or adapted to the research question. A clearly described search strategy for reviewing this literature is an indicator of the rigor you need to apply to the process of conceptualizing the problem [29,30]. The starting point is to compare various overarching conceptual models based on the power each may have to fully account for all the elements of the problem as you have chosen to define it. The introduction section of this paper offered examples from the 1997-2008 decade, and more recent examples can be found in references [9,31-34]. It is essential to justify your choice of a pre-existing conceptual model as it relates to the key features of your research problem. Alternatively, you may conclude that no pre-existing, cross-cutting conceptual models are adequate to explore this problem. This judgment also requires justification, and it opens the way for thought experiments about options for combining and elaborating the particular conceptual models that you have previously acknowledged. In one example, an investigation of a novel and under-researched technology in health care was able to proceed by integrating concepts of evidence-based treatment (from health science) and technology affordances (from information science) into a new conceptual model of therapeutic affordances of social media [35].

### Schematize the Chosen Conceptual Model of the Research Problem

Making a schematic representation of your chosen conceptual model captures and refines the thought processes behind your choice. A visual artifact in the form of a diagram, motif, map or other type of figure (eg, a foil or straw man) can be used for reacting to and testing the thinking about a problem, to guide collaboration, and to assess research progress and outcomes. An example of schematizing a health informatics conceptual model to represent the relationships among health information technology characteristics is the Health Information Technology Reference-based Evaluation Framework [36]. A second example illustrates the temporal dimensions of five measures of health information system adoption in the Clinical Adoption Meta Model [37].

The challenge for every health informatics researcher is to think deeply about an apt way to visualize the specific problem space. Part of the contribution that your research makes to the field is determined by the originality you show in this step. Questions to consider include: How does the visualization of your conceptual model position the information science and the health science elements of the problem (eg, side-by-side versus above and below)? Does the level of detail match the intended level of investigation (eg, evaluating the impact of a policy may need to represent issues at a macro level or assessing software functionality may need a finer grained picture)? Does it leave too much to be inferred (eg, not indicating the direction in which a multi-part image should be "read") or use conventions in an unconventional way (eg, using the colors red for "go" and green for "stop")?

The schema also needs to be interpreted in words that explain it to someone who is unfamiliar with the research problem or who does not have access to the graphic. It should be clear why you have chosen the visual representations you are using to represent the key entities and relationships that your problem involves. For example, a study of unfulfilled and unrecognized or hidden health information needs explained the research framework using the graphic image and written analogy of an iceberg [38].

Your first attempt to represent the entities and relationships in your research problem using a Venn diagram, matrix, or flowchart may not suffice to give adequate detail or insight into the problem space. The visualization of knowledge is a field of study in its own right, and you may find it helpful to consult general works [39,40]. Although the visualization of data has become an active health informatics research area [41,42], the visualization of concepts is a very different order of activity.

### Seek Critical Feedback on the Conceptual Model From Multiple Perspectives

Your conceptual model may appear completely sensible from your point of view. However, at this point in development, you should be thinking of your conceptual model as a communication tool. This tool should help you engage other people who are direct and indirect stakeholders in your research. Thus, stakeholders with other perspectives need to test its communicative power to assure you that it is making sense of the problem.

A conceptual model in health informatics research should pass the "goodness of fit" test for domain experts in both information science and health science. This is an informal but important step where you seek critique of your conceptual model from others who are at a distance from your research question. Their reactions allow you to refine and strengthen the supporting arguments for your conceptual model as needed. You are looking for toughness; now is the time to establish whether your conceptual model will stand up to scrutiny and be persuasive in a room full of either clinical specialists or computer scientists.

For a researcher in training, often the best way to ensure access to this kind of feedback is to make sure that the supervisory or advisory committee comprises people who bring information science and health science perspectives. Alternatively, the researcher needs to find suitable critical colleagues by tapping into networks of clinicians and researchers within the health and biomedicine community, and into networks of industry experts and researchers within the information science and technology community. Organizational mentoring programs, professional associations, or scientific societies can provide access to networks appropriate to the study.

If the research involves human participants, another method of seeking feedback on a conceptual model is to engage actively with prospective participants including patients and consumers. For example, Belanger et al. advocated involving patients from the inception of a research project centered on electronic health records [43].

### Allow the Conceptual Model to Influence the Research Design

There must be a close connection between your conceptual model and research design. In a different field of health research, the alignment was expressed as follows:

The CF (conceptual framework) provided the basis for decisions about the development of a mixed-method research design and data collection measures. For each construct of interest, we determined the most suitable approach to collecting information. We arranged the constructs into two categories: those where validated quantitative measures were available (...); and second, those that were best suited for exploratory/qualitative methods.

18

This is a good example of allowing the conceptual model to influence the research design, including the selection of the research procedures and outcome measures (for quantitative research) or themes (for qualitative research). Feasibility factors also influence research design, for example, access to sites for field studies, available funding and human resources, the exigencies of ethics approval, and time constraints. However, if any of these factors undermine the conceptual model to any great extent, then the research question and conceptual model need to be reformulated.

### Revisit the Conceptual Model in Light of the Research Findings

Your conceptual model is what guides the approach you take to explore a real-world research problem. The corollary is that through exploring this problem, inevitably you are testing your conceptual model for its usefulness. While you are analyzing and discussing findings from your research project, you need to reflect on whether and how these findings support your initial conceptual model, identify where the conceptual model may need to be modified, and consider broader circumstances where the conceptual model may prove useful. This is an iterative and experiential process that stretches your thinking as your conceptual model "evolves and develops until it becomes refined and burnished, to emerge as a robust outcome of the research" [44].

In health informatics research, the gap between health sciences and information sciences that must be bridged by this step is substantial.

In more mature fields such as medicine, it is standard practice, even mandatory, to conduct empirical research to evaluate the efficacy of proposed new practices prior to advocating their use (...). However in IS design research, it is often sufficient for researchers to argue on logical or theoretical grounds that their approach is effective.

45

How your health informatics conceptual model is tested depends on the research design appropriate to your study. In one example, thematic analysis of interview data is the basis for revising an initial conceptual model of health professionals’ mobile health use [46]. Examples where structured survey methods are the basis for revisions to initial conceptual models are found in studies of clinical information system success [47], and open access publishing use [48].

### Disseminate the Conceptual Model

The final step is to disseminate your conceptual model through formal presentation and publication in scholarly and scientific conferences and journals. It is important to give over the time and space to include the conceptual model among the publications that come out of your research, so that it is captured and available for others to refer to and build on. You can structure a paper or presentation about your conceptual model using steps one through six in this paper; or examples cited throughout this paper offer many other successful models for publishing a description of your conceptual model.

When you describe your work on a conceptual model you make a contribution to the theory of health informatics, because your conceptual model adds a new theoretical representation of the entities and relationships in a problem space. In disseminating your conceptual model you address an acknowledged need in the field. The absence of theory in health informatics diminishes its status as a field of knowledge [49]; whereas, enunciating your conceptual model advances the field [3].

There are many diverse forums where it is possible to disseminate your conceptual model. This is one advantage of the diffusion of published research in health informatics. Apart from those already mentioned in this paper, three further examples of particular health informatics conceptual models published in recent years illustrate that it may be appropriate to place your work in conferences and journals in information science [50], in health science [51], and in mainstream health informatics [52].

## Discussion

### Benefits of Developing and Using Conceptual Models

This paper outlines current challenges in developing a conceptual model that integrates the information science aspects and the health science aspects of a health sector information and communication technology problem. The effort to overcome these challenges can yield important benefits that are not only theoretical but also practical. For the individual researcher, the conceptual model provides a persistent reminder of the defined entities and relationships that give shape and direction to their research plan and the interpretation of their findings. For teams comprising various disciplines in a collaborative research project, the conceptual model helps to share the related vocabulary and reach agreement on the underlying constructs. For diverse researchers with different questions who are formulating their own approach or discussing their own findings, the conceptual model facilitates comparisons and cross-pollination of ideas. Beyond the research community, there are benefits from giving closer attention to health informatics conceptual models for three professional practice communities : health informatics practitioners, other health practitioners, and other IT practitioners.

The community of health informatics professionals can use conceptual models more overtly to improve practice. By eliciting organizational input into conceptualizing implementation issues [13], they may be able to communicate and surmount notable problems of sustaining health information and communication technology applications [30]. For instance, making deliberate use of a socio-technical conceptual model can help to anticipate unintended consequences before these emerge during system implementation [53]. Building health informatics conceptual modeling skills can enhance training and professional development for roles such as chief information officers in health organizations, research and development managers in health technology companies, and health informatics experts in large consulting firms. For example, health informaticians in such roles may benefit from working with conceptual models to explain and deliver the business value of information technology in healthcare [54].

Health professionals who are not information scientists can use conceptual models to facilitate inter-professional practice, exert collegial influence, and advance professional ethics in their work in an increasingly technological sector. For the individual health professional, making explicit the conceptual models you use in your professional practice enables you to integrate these more strongly into planning for new work practices during periods of technology change and adoption [55]. In working with colleagues in your profession, your ability to communicate conceptual models that frame health information and communication technology projects can position you as a leader and facilitator in the design and oversight of such projects [36]. In addition, the way each health profession expresses its ethical commitment to the safety and quality of care can be subtly different. When you clarify the conceptual models that you apply in health information technology projects that involve your own patients and/or clients in the settings where you provide care for them [56], you express more deeply the way you think about their needs and how technology interventions might address these. In doing so, you contribute to broadening the discourse within your profession about the ethical practice of clinical informatics.

Information professionals who are not health scientists can use integrative conceptual models to access important opportunities for innovation in the health sector, to achieve advances on health informatics grand challenges, and to build prized expertise and strong partnerships. Because information technology in health is developing massively and is being adopted rapidly, the health sector offers numerous opportunities to apply emerging solutions. When you aim to transfer into the health sector the conceptual models that underlie solutions from non-health sectors, acknowledging and articulating these enables you to reflect on how they can be expected to have positive impacts and be sustainable in health [57]. Coming to terms with the way information science conceptual models relate to the key health domain conceptual models where you are applying solutions can make you more effective in solving higher order problems [58]. If you are able to relate to and communicate within the health sector on this conceptual level [59,60], you will have expertise that is critical for successful collaboration with highly educated and committed health professionals to bring about technological transformation.

### Conclusion

Our aim is to provide a representative selection of examples to accompany our suggestions for researchers who are new to the health informatics discipline. It is important for health informatics researchers to elucidate their hard-won conceptual modeling experience, even while recognizing that no conceptual model can ever be static or definitive. Sharing and comparing these foundations of knowledge can support good practice in research training (and in the commissioning, management, and review of research), and thereby can contribute to the evolution of better-formed and more strongly grounded health informatics research. Making explicit the defined entities and relationships in a health informatics research project facilitates deep engagement with cross-cutting problems, offers a way for researchers to be more effective, and enables research to have greater impact.
